# Biometric and structural ocular manifestations of Marfan syndrome

**DOI:** 10.1371/journal.pone.0183370

**Published:** 2017-09-20

**Authors:** Petra Gehle, Barbara Goergen, Daniel Pilger, Peter Ruokonen, Peter N. Robinson, Daniel J. Salchow

**Affiliations:** 1 Department of Cardiology, Charité –University Medicine Berlin, Augustenburger Platz 1, Berlin, Germany; 2 Department of Ophthalmology, Charité –University Medicine Berlin, Augustenburger Platz 1, Berlin, Germany; 3 Private Practice Zehlendorf, Berlin, Germany; 4 The Jackson Laboratory for Genomic Medicine, Farmington, United States of America; Bascom Palmer Eye Institute, UNITED STATES

## Abstract

**Background:**

To study biometric and structural ocular manifestations of Marfan syndrome (MFS).

**Methods:**

Observational, retrospective, comparative cohort study in a tertiary referral center on 285 MFS patients and 267 controls. Structural and biometric ocular characteristic were compared.

**Results:**

MFS eyes were longer (axial length 24.25 ± 1.74 mm versus 23.89 ± 1.31 mm, p < 0.001) and had a flatter cornea than control eyes (mean keratometry 41.78 ± 1.80 diopters (D) versus 43.05 ± 1.51 D, p < 0.001). Corneal astigmatism was greater and the central cornea was thinner in MFS eyes (530.14 ± 41.31 μm versus 547.02 ± 39.18 μm, p < 0.001). MFS eyes were more myopic than control eyes (spherical equivalent -2.16 ± 3.75 D versus -1.17 ± 2.58 D, p < 0.001). Visual acuity was reduced (0.13 ± 0.25 logMAR versus 0.05 ± 0.18 logMAR, p < 0.001) and intraocular pressure was lower in MFS eyes (14.6 ± 3.4 mmHg versus 15.1 ± 3.2 mmHg, p = 0.01). Iris transillumination defects (ITD) were significantly more common in MFS eyes (odds ratio for MFS in the presence of ITD, 3.7). Ectopia lentis (EL) was only present in MFS eyes (33.4%). History of retinal detachment was significantly more common in MFS eyes. Glaucoma was equally common in both groups.

**Conclusions:**

ITD and EL are most characteristic findings in MFS. ITD and corneal curvature should be studied as diagnostic criteria for MFS. Visual acuity is reduced in MFS. MFS patients need regular eye exams to identify serious ocular complications.

## Introduction

Marfan syndrome (MFS), an autosomal dominant connective tissue disorder, is caused by mutations in the fibrillin-1 gene (*FBN1*).[1, 2] Fibrillin-1 is a major constituent of microfibrils, involved in elastic fiber formation.[3] Manifestations of MFS affect multiple organ systems including skeletal muscle, skin, the cardiovascular system (e.g. aortic dissection) and the eye.[4] Up to 54% of MFS patients have major ocular involvement,[5] and ocular findings may be crucial in establishing the diagnosis.

Diagnostic criteria were last updated in 2010 (Ghent 2 criteria).[6] In these criteria, ectopia lentis (EL) is a cardinal diagnostic feature. Other diagnostic ocular findings are down-slanting palpebral fissures, enophthalmos and myopia ≥ 3 diopters (D). Increased axial length (> 23.5 mm), flat cornea (< 41.5 D average corneal curvature), iris transillumination defects (ITD) and retinal detachment (RD) are common in MFS but not part of the Ghent 2 criteria.

Although descriptive studies on ocular manifestations of MFS are available, most are smaller series[7–10] focusing on biometric or structural findings without including a control group.[7, 8, 10]

To better define the ocular manifestations of MFS and to study which parameters may be of diagnostic value, we analyzed ocular biometric and structural findings in a large cohort of MFS patients and compared them to a control group.

## Materials and methods

### Study population

Records of patients presenting to the interdisciplinary Marfan clinic at Charité-Unversity Medicine Berlin over a period of four years (December 2009 to December 2013) were reviewed. The institutional review board approved the study and waived the need for study subjects to consent for their records to be used in this study. The data were collected and analyzed anonymously.

### Diagnosis of Marfan syndrome

A complete medical and family history was obtained. Patients received a skeletal exam, a cardiologic evaluation including a trans-thoracic echocardiogram, a genetic analysis and a complete ophthalmologic exam.

MFS was diagnosed according to the revised Ghent 2 criteria.[6] Patients with MFS were included in the study group, patients with a connective tissue disorder other than MFS were excluded. Patients without MFS and other hereditary connective tissue disorders were included in the control group.

### Ophthalmological examination

Best-corrected visual acuity, objective refraction (Humphrey Automatic Refractor 599, Carl Zeiss Meditec Inc., Dublin, CA, USA), and manifest refraction (if necessary) were measured. Intraocular pressure (IOP) was measured with non-contact tonometry (NCT, Nidek NT-530, Nidek Co. Ltd., Aichi, Japan), and confirmed with Goldmann applanation tonometry if NCT was unreliable or outside the normal range. A slitlamp exam of the anterior segment was done before and after pupil dilation. EL was diagnosed if the edge of the lens was visible after pupil dilation pupil (with neosynephrine 2.5% and tropicamide 1% eye drops). Dilated fundus examination was performed using indirect ophthalmoloscopy.

### Biometry

Central corneal thickness (CCT), axial length (AL), aqueous depth (AD, the distance between the posterior corneal surface and anterior lens vertex), anterior chamber depth (ACD, the distance between anterior corneal vertex and anterior lens vertex), and lens thickness (LT) were measured.

Average corneal curvature (K*med*) and corneal astigmatism (difference between maximal and minimal corneal curvature) were assessed using keratometry. Instruments used for biometry were the IOL Master™ (software version V.3.01, Carl Zeiss AG, Oberkochen, Germany), the LENSTAR LS 900® (Haag-Streit Holding AG, Könitz, Switzerland), the ORBSCAN II® (Bausch & Lomb, Rochester, NY, USA) and the GALILEI™ V5.2.1 (Ziemer Ophthalmic Systems AG, Port, Switzerland).

### Statistical analysis

Analysis was performed with SPSS 22 (IBM, Armonk, NY, USA). Chi-square test or Fisher's exact test were used for categorical variables, the t-test was for continuous variables with normal distribution, the Mann-Whitney U-test for those without normal distribution. Due to ethical concerns, the institutional review board (IRB) of Charité –University Medicine Berlin (Campus Charité Mitte, Charitéplatz 1, 10117 Berlin, Germany; email: ethikkommission@charite.de) does not allow us to share the patient data publicly.

## Results

### Study population

Of 624 patients, 285 had MFS and qualified as controls. There were 153 male and 132 female MFS patients, and 150 male and 117 female controls. MFS patients were older (33 ± 5.8 years, average ± standard deviation, range 1–75 years, versus 29.1 ± 14.9 years, range 3–76 years, p = 0.003), 37 MFS patients and 36 controls were younger than 15 years.

### Biometry

MFS eyes were longer than control eyes (Table 1).

**Table 1:** Biometry, dimensions and refractive error of eyes of Marfan patients and control eyes.

	Marfan	Control	P-Value
**AL (mm)**^**1**^	24.25 ± 1.74	23.89 ± 1.31	< 0.001
**AD (mm)**^**2**^	2.95 ± 0.37	3.06 ± 0.31	0.016
**ACD (mm)**^**2**^	3.38 ± 0.44	3.50 ± 0.38	< 0.001
**LT (mm)**^**2**^	4.01 ± 0.40	3.82 ± 0.42	< 0.001
**K*med* (D)**	41.78 ± 1.80	43.05 ± 1.51	< 0.001
**AST (D)**	1.23 ± 1.01	1.03 ± 0.74	0.015
**CCT (micron)**	530.14 ± 41.31	547.02 ± 39.18	< 0.001
**SE (D)**^**1**^^,^^**2**^	-2.16 ± 3.75	-1.17 ± 2.58	< 0.001

AL—axial length, AD—aqueous depth, ACD—anterior chamber depth, LT—lens thickness, K*med* (D)—mean corneal curvature in diopters, AST—corneal astigmatism, CCT–central corneal thickness, SE—spherical equivalent in diopters. Values are mean ± standard deviation.

1 –eyes with a history of retinal detachment repair were excluded from analysis

2 –only phakic eyes were analyzed

MFS eyes with EL were longer than those without (24.50 ± 2.06 mm versus 24.03 ± 1.33 mm, p = 0.0007). Anterior chamber depth was reduced in MFS eyes (Table 1), but comparable in MFS eyes with EL than in those without (AD, 3.01 ± 0.37 mm versus 2.94 ± 0.39 mm, p = 0.081; ACD, 3.43 ± 0.48 mm versus 3.38 ± 0.45 mm, p = 0.357). Lens thickness (LT) was increased in MFS eyes, but comparable in MFS eyes with and without EL (p = 0.221).

### Cornea

The cornea was flatter and corneal astigmatism higher in MFS eyes (Table 1). In 46.4% of MFS patients K*med* was < 41.5 D in at least one eye, compared with 18.7% of control patients (p < 0.001, odds ratio (OR) for MFS 3.8, 95%CI 2.5–5.6).

MFS eyes with EL had a flatter cornea (K*med* 41.13 ± 1.79 D versus 42.11 ± 1.66 D, p = 0.001) and higher corneal astigmatism (1.63 ± 1.15 D versus 1.00 ± 0.90 D, p < 0.001) than MFS eyes without EL.

The central cornea was thinner in MFS eyes compared with control eyes (Table 1), and thicker in MFS eyes with EL than in those without (535.99 ± 43.69 μm versus 525.31 ± 39.74 μm, p = 0.025).

### Refractive error

MFS eyes were significantly more myopic than control eyes (Table 1). EL was associated with higher myopia in MFS eyes (SE, -2.60 ± 4.78 D versus -1.76 ± 3.38 D, p < 0.001). Myopia was more common in MFS patients and eyes. Myopic patients were more likely to have MFS (Table 2).

**Table 2:** Frequencies and odds ratio for different degrees of myopia as a diagnostic criterion for Marfan syndrome. Odds ratios (OR) are adjusted for gender and age. 95% CI = 95% confidence interval. If only one eye of a patient fulfilled the criterion, the patient was included in the respective category. Aphakic, pseudophakic and eyes with a history of retinal detachment repair were excluded. (D = diopters).

	MFS Patients	Control Patients	OR	95% CI
**Myopia > 3 D**	80 (31.4%)	45 (19.1%)	1.9 *	1.3–3.0
**Myopia > 0.75 D**	174 (67.4%)	113 (47.3%)	2.3 *	1.6–3.3

Statistically significant differences are marked with an asterisk.

Of the MFS patients with myopia > 3 D in at least one eye, 57.5% had EL. AL in MFS eyes with myopia > 3 D was 25.21 ± 1.82 mm (25.91 ± 1.52 mm in control eyes with myopia > 3 D, p = 0.53) and 23.82 ± 1.31 mm in MFS eyes without myopia (p < 0.001).

### Visual acuity

MFS eyes had worse visual acuity (VA) than control eyes (0.13 ± 0.25 logMAR versus 0.05 ± 0.18, p < 0.001). MFS eyes with EL had worse VA than those without (0.21 ± 0.34 logMAR versus 0.065 ± 0.17, p < 0.001). After surgery for EL, VA was better in pseudophakic (0.16 ± 0.19 logMAR) than in aphakic MFS eyes (0.58 ± 0.67 logMAR, p = 0.005).

### Intraocular pressure

IOP was lower in MFS eyes (14.6 ± 3.4 mmHg versus 15.1 ± 3.2 mmHg in control eyes, p = 0.01). 4.9% of MFS patients had IOP ≥ 21mmHg in at least one eye compared with 5.3% of controls (p = 0.089), the IOP in MFS eyes with and without EL was comparable (p = 0.721).

### Structural findings

Structural findings are summarized in Table 3.

**Table 3:** Structural ocular findings in patients with Marfan syndrome (MFS) and controls. Total = number of patients with documented findings for the respective category, unilat. = unilateral, bilat. = bilateral.

Ocular Structure	MFS Patients	Controls	Pathology
	(normal/total)	(normal/total)	(MFS Patients/Controls)
External/Eyelids	268/274	256/260	Ptosis (unilat.) 1/1
			Milia (bilat.) 1/0
			Blepharitis/Meibom dysfunction 3/2
			Hordeolum 1/0
			Blepharospasm 0/1
Conjunctiva	273/278	257/261	Injection 2^1^/4^1^ (bilat. 0/2)
			Sliicone granuloma 1^2^/0
			Melanosis (bliat.) 0/1
			Pterygium (unilat.) 1/0
			Cystic bleb after trabeculectomy 2/0
Cornea	269/279	258/261	Endothelial precipitates (bilat.) 1^1^/0
			Opacities 3^1^/1^1^ (bilat. 2/0)
			Scar (unilat.) 0/1^1^
			Unspecified keratopathy 1^1^/1^1^
			Cornea guttata (bilat.) 3/0
			Corneal transplant (unilat.) 1^1^/0
			Cornea verticillata (unilat.) 1^1^/0
			Arcus senilis (bilat.) 1/0
			Krukenberg spindles (bilat.) 2/0
Anterior Chamber	277/278	260/260	Shallow anterior chamber (bilat.) 1/0
Iris	191/277	236/260	Transillumination defects^3^ 65/23
			Iridodonesis 10/0
			Iridectomy 3^1^/0
			Synechia 0/1^1^
Pupil	267/273	260/267	Corectopia 3^4^/1
			Miotic pupil 2/0
Vitreous	14/21	9/9	Opacities 4/0 (bilat. 3/0)
Optic Disc	248/271	235/251	Excavation 5/6 (bilat. 4/5)
			Blurred margins 3/6 (bilat. 2/5)
			Tilted disc 12/11 (bilat. 9/8)
			Makropapilla (bilat.) 3/3
			Pale disc (unilat.) 1^1^/0
Macula	252/272	247/257	Blunt reflex 5/6 (bilat. 4/4)
			Hemorrhage (unilat.) 1/0
			Unspecified maculopathy 3/1 (bilat. 2/1)
			Pigmentary changes 11/3 (bilat. 6/2)
			Macular hole (unilat.) 0/1
			Scar (unilat. 1/1
Retina	246/272	247/257	Peripheral degeneration incl. myopic 18/7 (bilat. 12/7)
			Peripheral hemorrhages (bilat.) 0/1

1. no documented cause or reason for this finding

2. Status post retinal detachment repair with silicone oil tamponade

3. eyes with previous intraocular surgery or history of retinal detachment excluded from analysis

4. Status post vitrectomy for one MFS patient, status post trabeculectomy for two others

For some categories, more or less than one finding was documented per patient.

ITD (in eyes without previous intraocular surgery or retinal detachment) were more common in MFS eyes than in control eyes (p < 0.001, OR for MFS in the presence of ITD was 3.7, 95%CI 2.3–6.2). ITD were more common in MFS eyes without EL (21%) compared with control eyes (7.4%, p < 0.001). ITD were mostly bilateral (81.5% MFS patients, 72.6% controls). 39/112 (34.8%) MFS eyes with ITD also had EL, compared to 77/351 (21.9%) without ITD (p = 0.008).

Of 76 MFS patients with ITD, 2 had glaucoma, 2 had suspected glaucoma, one had suspected keratoconus, one had a history of central retinal artery occlusion, one had a phthitic eye after retinal detachment repair and one had a cataract. Of the 24 controls with ITD, one had glaucoma, one had suspected glaucoma suspicion, two had cataracts and one had a history of retinal detachment repair. Iridodonesis was found in MFS patients only, it was bilateral in 80% and associated with EL in 70%.

#### Lens

Normal lens findings were documented in 119/277 MFS patients and 243/261 controls. Thirteen MFS patients were bilaterally aphakic, one unilaterally (12 had been operated on for EL). There was one aphakic control patient, without a history of EL. Twenty-six MFS patients were pseudophakic (7 unilaterally, 19 bilaterally), 17 had a history of EL, in 7 the reason for pseudophakia was not identified, and 2 had had cataract surgery. One control had unilateral pseudophakia after cataract surgery. One MFS patient had a history of lens coloboma.

Twenty-four MFS patients (8.7%, 12 men) had cataracts, 5 of them unilaterally. Of the 16 controls (6.1%, seven men) with cataracts, 3 had unilateral cataracts. Cataracts were equally common in both groups (p = 0.263).

#### Ectopia lentis

EL was only noted in MFS patients. In 2 MFS patients, clinical examination was not possible; both had a history of EL however and were counted as EL. EL was present in 182/544 MFS eyes (33.4%). Of 101 MFS patients with EL (43 males, 36.4% of patients with information on lens position), it was bilateral in 81. Average age of patients with EL was 30.3 ± 15.4 years versus 34.5 ± 15.8 years of those without (p = 0.341). The lens was decentered superiorly in the majority of eyes (Table 4). One MFS patient had bilateral phakodonesis without EL.

**Table 4:** Direction of lens subluxation in eyes of Marfan syndrome patients with ectopia lentis (n = 101 patients, 182 eyes).

Direction of Lens Subluxation	Number (percent)
Superior	74 (79.6%)
Superonasal	9 (9.7%)
Superotemporal	1 (1.1%)
Nasal	5 (5.4%)
Temporal	1 (1.1%)
Inferior	2 (2.2%)
Vitreous cavity	1 (1.1%)
Not specified	89
*Total*	*182 (100%)*

#### Ocular fundus

257/272 MFS patients and 255/257 controls had bilaterally normal fundi. Two MFS eyes had a choroidal nevus. Two highly myopic MFS patients had bilaterally "myopic fundi", another male MFS patient had a “myopic fundus” in a moderately myopic eye. One control eye had myelinated nerve fibers, another “tortuous retinal vessels”.

#### Retinal detachment

Ten MFS patients (3.7%, 36.4 ± 15.5 years, eight men) and two male controls (0.8%, 9 and 55 years old) had a history of retinal detachment (RD; p = 0.026). The risk for RD was increased in MFS patients (OR 2.81; 95%CI 1.0–22.2; p = 0.049; adjusted for gender and age). The RD was bilateral in 6 and unilateral in 2 MFS patients. In 2 MFS patients, information on laterality was not available. One control had bilateral and the other a unilateral RD. Of the MFS patients with RD, 7 had EL and 6 had previous lens surgery. Visual acuity of MFS eyes with RD was 0.61 ± 0.84 logMAR compared with 0.11 ± 0.21 of MFS eyes without (p < 0.001). AL of MFS eyes with RD was increased (28.6 ± 3.8 mm versus 24.1 ± 1.50 mm for MFS eyes without RD, p < 0.001).

#### Glaucoma

Eight MFS patients (49.6 ± 11.8 years, 4 men) had glaucoma, 4 also had EL and 2 had a history of RD. MFS eyes with glaucoma had an insignificantly thinner cornea (CCT, 515 ± 63 μm) than those without (530 ± 40 μm, p = 0.218). Ten MFS patients (39.6 ± 21.2 years, 7 men) were glaucoma suspects, 3 of them had EL, one had a history of RD.

Six controls (49.0 ± 14.1 years, five men) had glaucoma, one also had peripheral retinal degenerations, glaucoma prevalence was comparable in both study groups. Five controls were glaucoma suspects (38 ± 14.9 years, 3 men), making suspected glaucoma equally common in both groups.

#### Other diagnoses

One MFS patient had a history of unilateral branch retinal vein occlusion, and 2 had a history of unilateral central retinal vein occlusion. One control had trochlear palsy, another had a persisting hyaloid artery and 2 had congenital unilateral toxoplasmosis (one had a macular hole, the other a macular scar).

## Discussion

In this study, we compare 285 MFS patients to 267 controls with regard to biometric and structural ocular findings. To our knowledge, this is the largest comprehensive study on ocular manifestations of MFS including a control group.

### Biometry

AL in MFS eyes was increased, confirming earlier studies.[7, 8, 10–12] AL of control eyes was comparable with that of normal adults.[13–15] We confirmed that MFS eyes with EL are longer than those without.[7, 12, 16] It is conceivable that eyes with EL were more severely affected, which is supported by the finding that ITD and RD were more common in eyes with EL.

Despite the increased AL in MFS eyes, AD and ACD were decreased, agreeing with the findings of Konradsen et al.[17] Although statistically significant, the difference between MFS and control eyes was small and unlikely to have clinical importance.

MFS eyes had increased LT. Like others,[7, 17] we did not find a significant difference in LT between MFS eyes with EL and those without. One may hypothesize that significant changes in LT only occur in more severe degrees of EL.

### Cornea

Possible reasons for reduced corneal curvature in MFS found in this and previous studies[8, 18, 19] have been discussed elsewhere.[11] Konradsen and colleagues[9] reported that 38% of MFS eyes and 8% of control eyes had a K*med* < 41.5 D. In our study, 46.4% of MFS eyes and 19.6% of control eyes met this criterion, suggesting that corneal curvature deserves further evaluation as a diagnostic criterion for MFS. Because a flatter cornea and increased corneal astigmatism is associated with EL,[8] keratometry should be performed on all patients with MFS. It should be studied if children with MFS and abnormal corneal parameters are at increased risk to develop EL.

The central cornea thinner in MFS eyes compared to control eyes, which has been described before.[16, 18, 19] Although not pathologic in itself, this may have clinical relevance as a risk factor for primary open-angle glaucoma.[20] Despite decreased corneal thickness, keratoconus was not noted and does not seem to have an increased prevalence in MFS patients.[11, 19]

### Refractive error

Although not specific, myopia > 3 D is part of the systemic score for MFS.[6] We found that MFS eyes were significantly more myopic than control eyes, whereas the prevalence of myopia and the mean refractive error of controls were comparable with the general German population.[21] Myopia > 3 D increased the likelihood for MFS almost twofold in our predominantly Caucasian study population. The myopia was considerably milder in our MFS patients compared with those of a recent study.[8] One should consider that the prevalence of myopia varies markedly between races.[21, 22] Higher degrees of myopia in MFS patients should raise the suspicion for EL and increased axial length, a risk factor for retinal complications.

### Visual acuity

There are several factors contributing to the decreased visual acuity in MFS[7, 10, 16] including EL, myopia, aphakia and retinal complications. Maumenee[11] reported a visual acuity of 20/40 (0.3 logMAR) or better in 70.5% of MFS eyes, compared with 91.8% of MFS eyes in our study. Possible reasons for this difference include improved diagnostic and therapeutic options today as well as different study group composition. EL is associated with reduced visual acuity in MFS eyes.[7, 16] After surgery for EL, pseudophakic MFS eyes had better visual acuity than aphakic MFS eyes. Apart from aphakia itself, amblyopia and more severe ocular involvement in aphakic eyes may contribute to this finding.

### Intraocular pressure

MFS eyes had lower IOP than control eyes, but the absolute difference was likely not clinically significant. To some degree, the thinner central cornea in MFS patients may have caused underestimation of IOP. In contrast to previous reports,[7, 16] we did not find the IOP to be affected by EL.

### Structural findings

Pathologic external, adnexal and conjunctival findings were sporadic in both groups.

Although corneal findings were more common in MFS eyes, it is difficult to draw any conclusions because of the low overall number in each subcategory.

Primary ITD and iridodonesis (in eyes without previous intraocular surgery) were more common in MFS patients than in controls. Maumenee reported ITD in approximately 10% of MFS patients,[11] which is lower than our frequency. ITD are not pathognomonic for MFS, they can occur in pseudoexfoliation syndrome (PEX), in pigment dispersion syndrome (PDS), after ocular surgery, trauma and in intraocular inflammation (e.g. herpetic uveitis). None of these diagnoses were present in subjects with ITD. With an OR of 3.7, ITD should be considered as a diagnostic criterion for MFS.

Secondary iris findings such as iridectomies are not characteristic for MFS. They probably reflect the higher number of previous intraocular surgeries in MFS eyes.

#### Lens

EL is a major diagnostic criterion for MFS and was highly predictive of MFS in our study. However, it is not pathognomonic for MFS and may be associated with other disorders including ocular trauma, infection, inflammation and tumors. Homocystinuria,[23] dominant familial ectopia lentis and other genetic disorders may also cause EL.[24, 25]

Our rate of EL was comparable with recent studies from Sweden[10] and the UK,[26] but significantly lower than the 60–79% reported in earlier investigations.[7, 11, 23] Unlike other investigators,[7] we did not categorize phakodonesis as EL. We recommend standardization of criteria for EL, because of its importance for the diagnosis of MFS. Like previous reports,[11, 23] we found that the lens was subluxated superiorly in most eyes with EL. The pathomechanism of EL in MFS has been discussed in detail elsewhere.[4, 5, 11, 27]

The prevalence of cataracts was comparable between MFS patients and controls, but MFS patients with cataracts tended to be younger. It remains to be seen whether there is an association between MFS and early cataracts. Pseudophakia and aphakia were significantly more common in MFS patients than in controls. In most cases, EL was the reason for lens surgery.

#### Vitreous

The vitreous was most likely not systematically examined, conclusions on the prevalence of vitreous changes are therefore problematic. Vitreous liquefaction has been reported in MFS.[28]

#### Ocular fundus and retina

Myopia-related ocular fundus findings were common in MFS. Peripheral retinal degenerative changes were noted more than twice as often in MFS eyes compared to control eyes. Pigmentary macular changes may also represent findings secondary to higher degrees of myopia in MFS eyes.

In MFS, the risk for RD was increased, in 60% of MFS patients with RD, it was bilateral. RD is a sight-threatening complication, reflected by the decreased visual acuity in eyes with a history of RD. The pathomechanism and treatment of RD in MFS eyes has been discussed in detail.[28–32]

While some mostly older studies reported RD in 8%[11] of MFS patients and 9–19% of MFS eyes,[7, 23] more recent studies including this one found lower frequencies.[5, 10, 33] Most of our MFS patients with RD had a history of EL or lens surgery. Also, MFS eyes with a history of RD were significantly longer than MFS eyes without. We recommend regular ocular fundus examinations for all MFS patients, particularly those with a history of EL, lens surgery or increased AL.

#### Glaucoma

Decreased central corneal thickness is known risk factor for the conversion from ocular hypertension to glaucoma.[20] Although the central cornea of MFS eyes was thinner, glaucoma was not more common in than in controls. Some studies suggest that glaucoma may be more prevalent in MFS than in the general population,[5, 34] with open-angle glaucoma being the most common type, followed by secondary glaucoma.[34] It appears prudent to evaluate MFS patients for glaucoma.

There are limitations to this study. First, documentation was not standardized and sometimes incomplete. Second, different instruments were used for biometry during the course of the study, reflecting the technological progress during the study period. Although a connective disorder was ruled out in our controls, they probably do not represent the general population. We believe that inclusion of a control group facilitates interpretation of the findings. When comparing our results with earlier studies, one should consider that diagnostic criteria for MFS continue to evolve, which affects study group composition.[35] We used the current (Ghent 2) criteria for the diagnosis of MFS.

MFS patients were older than controls. Where appropriate, statistical analysis corrected for age. Finally, cycloplegia was not routinely documented in children in this study, a lack thereof may have caused overestimation of myopia in some children. However, the number of MFS patients and controls under 15 years of age was similar, so this potential effect was probably symmetric between the groups.

In summary, MFS patients are at increased risk for myopia, EL, RD, and reduced visual acuity. Decreased corneal curvature and ITD should be considered as diagnostic criteria. The knowledge of ocular manifestations of MFS is essential for early detection and to avoid complications which may permanently reduce vision.
